# JC Virus Agnogene Regulates Histone-Modifying Enzymes via PML-NBs: Transcriptomics in VLP-Expressing Cells

**DOI:** 10.3390/v17101399

**Published:** 2025-10-21

**Authors:** Yukiko Shishido-Hara, Takeshi Yaoi

**Affiliations:** 1 Department of Diagnostic Pathology, Kindai University Hospital, Osaka-Sayama 589-8511, Japan; 2 Department of Pathology and Applied Neurobiology, Kyoto Prefectural University of Medicine, Kyoto 602-8566, Japan; tyaoi@koto.kpu-m.ac.jp

**Keywords:** JC virus (JCV), promyelocytic leukemia (PML), promyelocytic leukemia nuclear bodies (PML-NBs), agnogene, capsid proteins, histone modification, chromatin dynamics, virus-like particles (VLPs)

## Abstract

JC virus (JCV) replicates within the nuclei of glial cells in the human brain and causes progressive multifocal leukoencephalopathy. JCV possesses a small, circular, double-stranded DNA genome, divided into early and late protein-coding regions. The non-coding control region (NCCR) functions bidirectionally for both early and late genes, and the agnogene is located downstream of TCR and upstream of three capsid proteins in the late region. Previously, in cell culture systems, we demonstrated that these capsid proteins accumulate in intranuclear domains known as promyelocytic leukemia nuclear bodies (PML-NBs), where they assemble into virus-like particles (VLPs). To investigate the agnogene’s function, VLPs were formed in its presence or absence, and differential gene expression was analyzed using microarray technology. The results revealed altered expression of histone-modifying enzymes, including methyltransferases (EHMT1, PRMT7) and demethylases (KDM2B, KDM5C, KDM6B), as well as various kinases and phosphatases. Notably, CTDP1, which dephosphorylates the C-terminal domain of an RNA polymerase II subunit, was also differentially expressed. The changes were predominant in the presence of the agnogene. These findings indicate that the agnogene and/or its protein product likely influence epigenetic regulation associated with PML-NBs, which may influence cell cycle control. Consistently, in human brain tissue, JCV-infected glial cells displayed maintenance of a diploid chromosomal complement, likely through G2 arrest. The precise mechanism of this, however, remains to be elucidated.

## 1. Introduction

JC polyomavirus (JCV) infects most of the healthy human population [1,2,3]. Under conditions of immunosuppression, it can reactivate and cause progressive multifocal leukoencephalopathy, a devastating demyelinating disease of the central nervous system (CNS). Although its unique neuropenetrance remains poorly understood, in the early 2000s, it was suspected that the presence of a virus-specific neuroreceptor might underlie this phenomenon. It was found that terminal α2,6-linked sialic acid residues on glial cell surface membranes interact with JCV capsid proteins, facilitating cell entry via clathrin-coated pits [4]. However, this molecule was also found on the surface of other cell types, including lymphocytes and spleen and tonsil cells [5], indicating that its expression is not restricted to the nervous system. If so, an intranuclear mechanism may define the neuro-specific pathogenicity of JCV [6], and the presence of neuro-specific transcription factors is one possibility. Consequently, attention has increasingly shifted to intranuclear factors as potential determinants of JCV neuropathogenesis; nevertheless, the underlying mechanisms remain to be clarified.

JCV possesses a small, double-stranded DNA genome that shares approximately 70% sequence homology with simian virus 40 (SV40) and BK virus (BKV) [7]. Its genome comprises early and late coding regions; within the late region, the major capsid protein VP1 and the minor capsid proteins VP2 and VP3 are encoded downstream of the agnogene. The agnogene and its product, agnoprotein, are multifunctional: the former, in cis, suppresses the downstream capsid-protein expression [8], and the latter, in trans, plays divergent roles, including transcriptional control and maturation of virions. Previous studies have shown that the major and minor capsid proteins accumulate at discrete intranuclear sites known as promyelocytic leukemia nuclear bodies (PML-NBs), where virus-like particles (VLPs) are formed. Here, the abbreviation for promyelocytic leukemia (PML) is identical to that of progressive multifocal leukoencephalopathy [7,9,10]. Interestingly, in one study, VLPs formed in the presence of the agnogene were more uniform in size and appeared to exhibit greater pathogenicity. In contrast, VLPs were deformed and unlikely to be pathogenic in its absence [8]. An extensive analysis via structured illumination microscopy with higher resolution revealed that, in human brain tissue, JCV-infected cells exhibited well-developed PML-NBs [11,12], which were as large as approximately 1.0 μm in diameter, especially in the G2 phase [8]. JCV capsid proteins were located on the surface of the largest bodies, suggesting active viral replication on the PML-NB surface.

The role of PML-NBs in JCV-infected cells is not yet fully understood. Notably, in living cells in vitro, it has been reported that PML-NBs increase in quantity during the early S phase [13]. The number of PML-NBs is reduced in the G2 phase, but giant PML-NBs, measuring over 1 μm in diameter, appear. These are known to function in the process of heterochromatin remodeling [14,15], and it was found that JCV actively produces progeny within these bodies. However, there is a paucity of the literature focusing on the functions of PML-NBs in JCV-infected human brain tissues.

In this study, we analyzed the functions of PML-NBs in using a cellular model to produce JCV-like particles in the presence or absence of the agnogene. While the PML protein is essential for maintaining the structural integrity of PML-NBs, the localization of other constituent proteins is dynamic—shifting between PML-NBs and the nucleoplasm depending on the cellular state. Alterations in PML-NB components were examined via microarray analysis, and data reflecting the human brain tissues are discussed.

## 2. Materials and Methods

### 2.1. Human Brain Tissues

Human brain tissues affected by progressive multifocal leukoencephalopathy were obtained from autopsy [11,12] and were processed into formalin-fixed, paraffin-embedded (FFPE) blocks using standard protocols. Thin sections were deparaffinized, rehydrated, and then subjected to immunofluorescence staining, followed by analysis using laser confocal microscopy (Leica Microsystems, Wetzlar, Germany). In addition, the sections were analyzed using a three-color fluorescence in situ hybridization (FISH) technique, as described below.

### 2.2. Expression System to Produce JCV-like Particles (VLPs)

The JCV Tokyo-1 strain (GenBank accession number AF030085) was used to create expression vectors, as described previously [7]. The constructs referred to in earlier studies as AVP231-SRα and VP231-SRα are herein designated as WT/AVP231 and Dl-Mt/VP231, respectively [8]. In brief, polycistronic JCV genomic fragments were cloned downstream of the strong SRα promoter, a chimeric regulatory element composed of the SV40 promoter-enhancer and the long terminal repeat (LTR) of human T-cell leukemia virus type 1 (HTLV-1). The WT/AVP231 construct harbors a viral genomic fragment spanning nucleotides 275 to 2531, encompassing the complete coding sequences for the agnoprotein (71 amino acids; nt 275–490), VP2 (344 amino acids; nt 524–1558), VP3 (225 amino acids; nt 881–1558), and VP1 (354 amino acids; nt 1467–2531). In contrast, the Dl-Mt/VP231 construct contains a fragment from nucleotides 410 to 2531, encoding VP2, VP3, and VP1 but lacking a 135 bp region (nt 275–409) that includes the ATG initiation codon of the agnogene. These constructs are unique in that the resulting vector-derived RNAs possess multiple open reading frames (ORFs). Upon transfection into COS-7 cells, these RNAs underwent alternative splicing using the authentic viral splice donor and acceptor sites, thereby enabling the expression of the major capsid protein VP1 and the minor capsid proteins VP2 and VP3 in physiologically relevant ratios. Furthermore, the formation of virus-like particles (VLPs) was confirmed by electron microscopy. For further details, refer to previous publications [7,9].

### 2.3. Laser Scanning Confocal Microscopy (LSCM)

For LSCM, sections of FFPE human brain tissues were prepared as described below. Cells were cultured on poly-L-lysine-coated tissue culture glass slides (Falcon) and transfected using the constructs described above. Approximately 72 h post-transfection, the cells were washed with phosphate-buffered saline (PBS) and fixed in 4% paraformaldehyde in PBS for 15 min at room temperature. Following fixation, the cells were permeabilized with 0.5% Triton X-100 in PBS for 20 min at room temperature. After this, the cells were rinsed in PBS containing 0.05% Tween-20 and then blocked with PBS supplemented with 5% normal goat serum for 30 min at room temperature. Primary staining was performed by incubating the cells with an anti-VP1BC antibody [7,9] for 1 h, followed by washing in PBS with 0.05% Tween-20. Cells were then incubated with a secondary antibody conjugated to Alexa Fluor 568. For double immunostaining, cells were also incubated with an anti-PML antibody, followed by a secondary antibody conjugated to Alexa Fluor 488 (Molecular Probes, Invitrogen, Carlsbad, CA, USA). After washing in PBS, the samples were mounted using VectaShield (Vector Laboratories, Burlingame, CA). Fluorescence images were acquired with a TCS-SP confocal laser scanning microscope (Leica, Heidelberg, Germany).

### 2.4. Microarray Analysis

Total RNA was extracted from cells transfected with WT/AVP231, Dl-Mt/VP231, or an empty vector control. RNA quality and quantity were assessed using a NanoDrop 1000 spectrophotometer (Thermo Fisher Scientific Inc, Waltham, MA, USA) and an Agilent 2100 Bioanalyzer (Agilent Technologies, Santa Clara, CA, USA). For complementary RNA (cRNA) amplification and labeling, the Agilent Low Input Quick Amp Labeling Kit (Agilent Technologies, Santa Clara, CA, USA) was used according to the manufacturer’s instructions. Briefly, total RNA was reverse-transcribed into double-stranded cDNA using a poly (dT)-T7 promoter primer at 65 °C for 10 min. The reaction was then incubated at 40 °C for 2 h in the presence of 5× First Strand Buffer, 0.1 M DTT, 10 mM dNTP mix, and AffinityScript RNase Block Mix (Agilent Technologies, Santa Clara, CA, USA). The reverse-transcription enzyme was inactivated at 70 °C for 15 min, and the resulting cDNA served as a template for in vitro transcription to synthesize Cy3-labeled cRNA. The transcription reaction was carried out using 5× Transcription Buffer, 0.1 M DTT, NTP mix, T7 RNA polymerase, and Cyanine-3-CTP, followed by incubation at 40 °C for 2 h. Labeled cRNAs were purified using RNeasy Mini spin columns (QIAGEN, Venlo, The Netherlands) and eluted in 30 μL of nuclease-free water. The concentration of cRNA and the efficiency of Cy3 dye incorporation were evaluated using the NanoDrop 1000 and Agilent 2100 Bioanalyzer.

For hybridization, 0.60 μg of Cy3-labeled cRNA was fragmented and applied to Agilent SurePrint G3 Human GE v3 8 x 60K Microarrays (Design ID: 072363). Hybridization was performed at 65 °C for 17 h. Following this, the arrays were washed and scanned using the Agilent SureScan Microarray Scanner (Model G2600D) (Agilent Technologies, Santa Clara, CA, USA).

### 2.5. Evaluation of Differentially Expressed RNAs

Intensity values for each scanned feature were quantified using Agilent Feature Extraction Software version 11.5.1.1, which includes background subtraction. Only features flagged as having no errors (i.e., “Detected”) were included in the analysis. Features flagged as not positive, not significant, not uniform, not above background, saturated, or population outliers (i.e., “Not Detected” or “Compromised”) were excluded to ensure data quality. Signal intensities were normalized using the 75th percentile shift method implemented in Agilent GeneSpring software version 13.1.1. Differentially expressed genes were identified based on a fold-change threshold of 1.5 or greater for both upregulation and downregulation. The experiments up to this point were conducted at DNA Chip Research Inc. (Kawasaki, Japan).

Differentially expressed genes were further analyzed with BLAST version 2.16.0 for consideration of the sequence similarity rate between homo sapiens and the monkey used (the origin of the cells). Finally, we extracted genes that showed a more than a two-fold change in expression and performed GO analysis using public web tools, such as DAVID, https://davidbioinformatics.nih.gov/home.jsp (accessed in 1 February 2025), Metascape, https://metascape.org/gp/index.html#/main/step1 (accessed in 1 February 2025), and Enricher, https://maayanlab.cloud/Enrichr/ (accessed in 1 March 2025).

### 2.6. Three-Color FISH

Thin-sectioned FFPE human brain tissues were deparaffinized, dehydrated, and air-dried. Antigens were retrieved by autoclaving the sections in 1 mM EDTA solution (pH 8.0) for 20 min. The sections were then allowed to cool to room temperature, rinsed with distilled water, and dried. JCV (Tokyo-1 strain) DNA was biotinylated using the nick translation method and subsequently detected with streptavidin-Cy2 for probe labeling. In addition, chromosome-specific fluorescence in situ hybridization (FISH) probes were employed, a Cy3-labeled one for human chromosome 8 and a digoxigenin-labeled one for chromosome 18, the latter visualized using anti-digoxigenin-Cy5. All three probes were applied to the prepared tissue sections. Denaturation was performed on a hot plate at 90 °C for 10 min, followed by hybridization at 37 °C overnight. Post-hybridization washes included incubation in 50% formamide/2 × SSC at 37 °C for 20 min and in 1 × SSC at room temperature for 10 min. Sections were then incubated with streptavidin-Cy2 and anti-digoxigenin-Cy5 to detect the corresponding labeled probes. Fluorescence signals were captured and analyzed using the Leica CW-4000 imaging system. The experiments were conducted at Chromosome Science Lab (Sapporo, Japan).

### 2.7. Generative AI and AI-Assisted Technologies in the Writing Process

During the preparation of this work, the authors utilized ChatGPT-4o, DeepL, and Grammarly to assist with academic writing and proofreading. After using these tools, the authors reviewed and edited the content as needed, and they take full responsibility for the content of the publication.

## 3. Results

### 3.1. Development of PML-NBs in the Enlarged Nuclei of JCV-Infected Human Glial Cells

**Figure 1:**
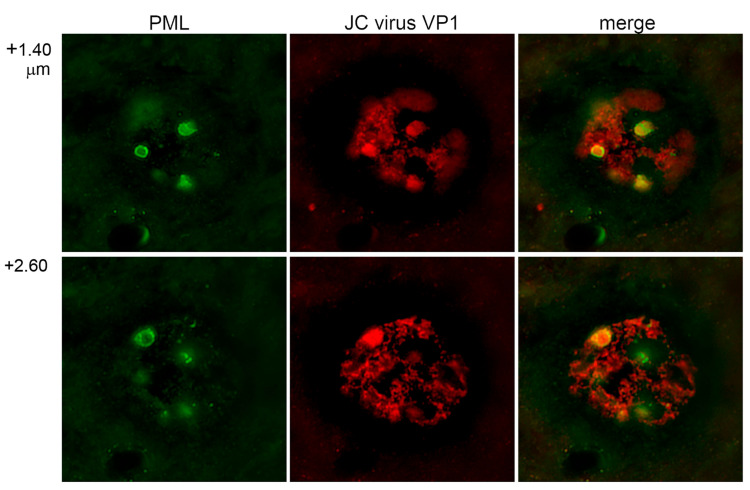
Localization of JCV VP1 to PML-NBs in infected human glial cells. LSCM images of glial cells from a human brain affected by progressive multifocal leukoencephalopathy. JCV VP1 was detected with Alexa Fluor 568 (red) and PML protein and Alexa Fluor 488 (green). The merged images demonstrate the co-localization of VP1 and PML consisting of nuclear bodies (PML-NBs). PML-NBs appeared as ring-shaped structures beneath the inner nuclear membrane. The largest bodies, typically observed in cells in the G2 phase, exhibited a prominent annular morphology in the optimal X-Y section. In the panels, “+1.40” and “+2.60” indicate the Z-axis coordinates.

We first examined the intranuclear localization of JCV capsid protein in human brain tissue obtained from an autopsied patient with progressive multifocal leukoencephalopathy. Immunofluorescence staining was performed using an antibody against the major JCV capsid protein VP1, conjugated to Alexa Fluor 568 (red), and an antibody against promyelocytic leukemia (PML) protein, a key component of PML nuclear bodies (PML-NBs), conjugated to Alexa Fluor 488 (green). Confocal microscopy revealed the presence of JCV-infected cells exhibiting marked nuclear enlargement, exceeding 180 μm in diameter (Figure 1). This was consistent with previous observations and statistical analysis [11]. The PML protein displayed a prominent punctate staining pattern, forming annular structures in two-dimensional images and spherical domains in three-dimensional reconstructions, indicative of PML-NB formation. Localization of VP1 to PML-NBs was confirmed (the merged panels in Figure 1). These findings corroborate earlier observations that PML-NBs are formed in JCV-infected cells, particularly in association with cell cycle progression from the S to the G2 phase [11]. It is notable that PML-NBs attain their maximal size during the latter phase, a phenomenon that is particularly pronounced under pathological conditions. These findings support our previous observations that JCV utilizes PML-NB-associated nuclear functions to facilitate the production of viral progeny [8].

### 3.2. Cellular Model of Intranuclear Inclusions with JCV-like Particles

**Figure 2:**
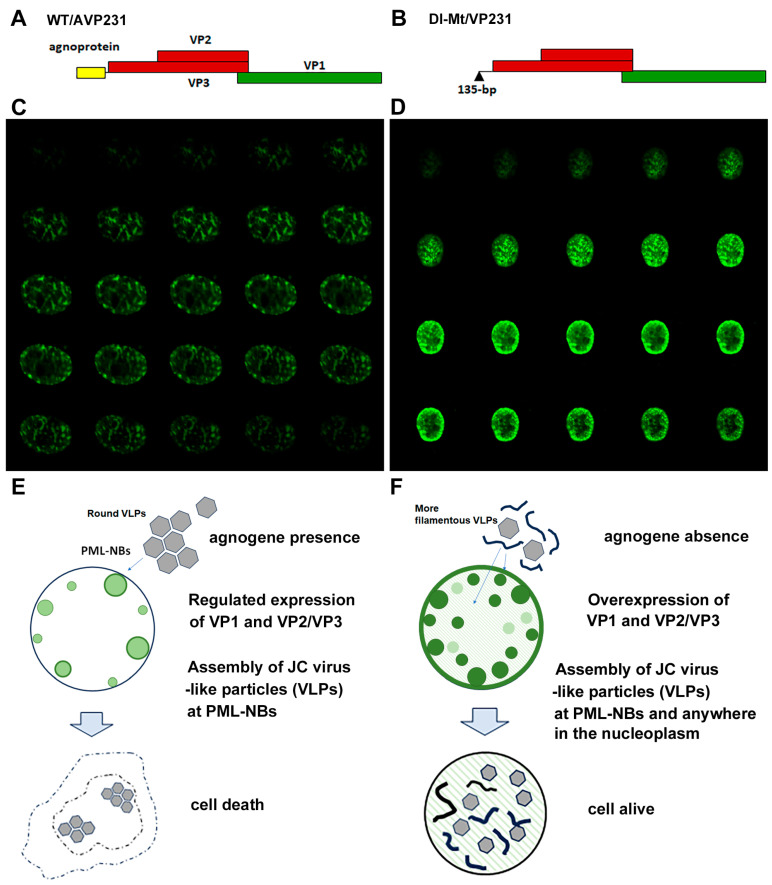
Expression constructs and intranuclear localization of JCV VP1. (**A**,**B**) A schematic representation of the expression vectors WT/AVP231 and Dl-Mt/VP231. WT/AVP231 includes the agnogene and all three capsid proteins (VP1, VP2, VP3), while Dl-Mt/VP231 lacks the 135 bp region including the agnogene start codon. (**C**,**D**) Confocal images of transfected COS-7 cells. WT/AVP231-transfected cells showed VP1 localization to discrete nuclear foci, indicating PML-NBs, whereas Dl-Mt/VP231-transfected cells displayed reduced association with PML-NBs with elevated VP1 expression levels. (**E**,**F**) Summary diagrams of previous electron microscopic analyses illustrating the morphology of virus-like particles (VLPs). WT/AVP231 led to the formation of uniform, spherical VLPs within PML-NBs, whereas Dl-Mt/VP231 resulted in irregular VLPs and filamentous structures.

Next, a eukaryotic expression system was utilized to investigate agnogene functions in the context of the PML-NBs [7,9]. Figure 2A,B schematically illustrate the expression of the constructs WT/AVP231 and Dl-Mt/VP231, in which a polycistronic viral genomic fragment is inserted into a cDNA expression vector: WT/AVP231 encodes the complete agnogene and the capsid proteins VP1, VP2, and VP3, while Dl-Mt/VP231 lacks a 135 bp segment from the 5′ end of the agnogene. Our previous studies demonstrated that transfection with WT/AVP231 resulted in translational repression of viral proteins, and VP1 exhibited distinct punctate localization beneath the nuclear envelope, consistent with targeting PML-NBs. In contrast, Dl-Mt/VP231 transfection resulted in elevated VP1 expression and diffuse intranuclear distribution, with minimal accumulation to PML-NBs [8]. The intranuclear localization of JCV VP1 in cells transfected with WT/AVP231 or Dl-Mt/VP231 is presented in Figure 2C,D, respectively. It was repeatedly shown that in the presence of the agnogene and/or its product, the expression level of VP1 was reduced, yet it restrictively localized to PML-NBs [8,9].

Through electron microscopic analysis, it was revealed that WT/AVP231-transfected cells formed uniform, spherical virus-like particles (VLPs) within PML-NBs and displayed prominent cytopathic changes. Dl-Mt/VP231-transfected cells contained variably sized VLPs and filamentous structures within enlarged but structurally intact nuclei. The results are schematically summarized in Figure 2E,F, and the details are reported in previous publications [8].

### 3.3. Microarray Analysis of JCV Capsid Proteins and PML-NB Dynamics

**Figure 3:**
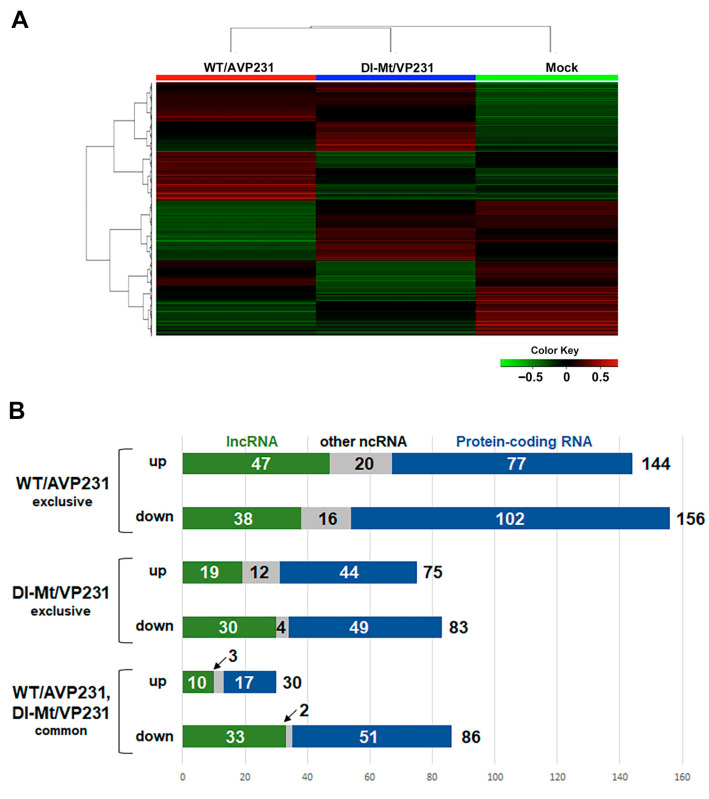
A heatmap of differentially expressed RNAs in transfected cells. (**A**) A heatmap showing the top 12,000 differentially expressed genes in cells transfected with WT/AVP231 or Dl-Mt/VP231, compared to mock-transfected controls. Expression patterns were distinct between the agnogene-positive and agnogene-negative conditions. (**B**) A bar graph summarizing the number of upregulated and downregulated transcripts (with a change greater than 2 folds) for each condition. WT/AVP231 induced more changes than Dl-Mt/VP231, with both conditions sharing some differentially expressed genes.

We next conducted a bulk transcriptome analysis through differential display with and without the agnogene. The aim was to clarify potentially PML-NB dynamics concerning the agnogene and VP1 distribution patterns. We analyzed the differential gene expression of cells transfected with WT/AVP231 or Dl-Mt/VP231, with mock-transfected cells serving as controls. The top 12,000 differentially expressed genes were visualized using a heat map, revealing mutually exclusive expression patterns between the two transfection groups, strongly implicating a functional role of the agnogene and/or its protein product (Figure 3A). Genes that exhibited a log_2_ fold change greater than 1 or less than −1 were subjected to further analysis. In WT/AVP231-transfected cells, 144 genes were upregulated and 156 were downregulated, compared to 75 upregulated and 83 downregulated genes in Dl-Mt/VP231-transfected cells. A smaller subset of transcripts exhibited shared expression changes under both conditions, with 30 genes being upregulated and 86 genes being downregulated. Approximately 60% of the differentially expressed RNAs were protein-coding, while the remainder were non-coding RNAs, including long non-coding RNAs (lncRNAs), which exhibited particularly pronounced expression variability. Expression profiles are summarized in bar graphs (Figure 3B), and the data are also presented in Supplementary Dataset S1.

### 3.4. Nuclear Gene Expression Signatures

**Figure 4:**
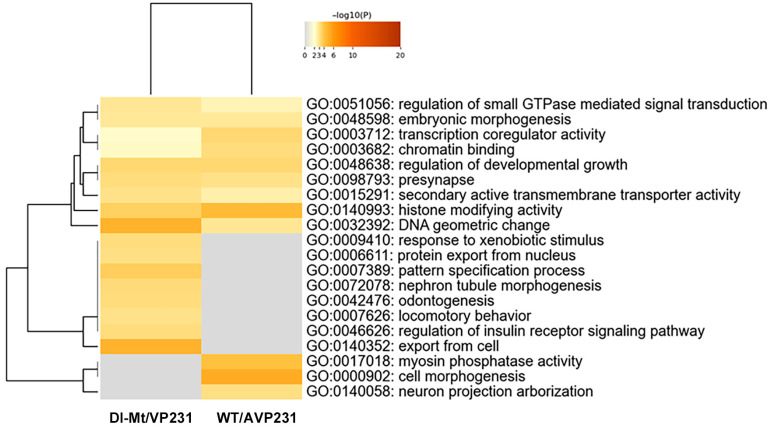
GO analysis of protein-coding RNAs in transfected cells. GO term enrichment (top 20 categories) based on differentially expressed protein-coding RNAs in WT/AVP231- and Dl-Mt/VP231-transfected cells, as determined by Metascape. Enriched GO terms included transcription coregulator activity (GO:0003712), chromatin binding (GO:0003682), histone-modifying activity (GO:0140993), and DNA geometric change (GO:0032392), suggesting nuclear regulatory roles for the agnogene.

Expression of protein-coding RNA was examined in more detail, and the numbers of differentially expressed nuclear proteins and non-nuclear proteins in WT/AVP231 (247 genes) and Dl-Mt/VP231 (161 genes) are summarized in Supplementary Dataset S2A. Gene Ontology (GO) analysis of protein-coding RNA was conducted using Metascape software. The data is presented in Figure 4, which shows that the top 20 enriched GO terms in both groups included transcription coregulator activity (GO:0003712), chromatin binding (GO:0003682), histone-modifying activity (GO:0140993), and DNA geometric change (GO:00032392). These terms highlight nuclear regulatory processes, suggesting that JCV infection affects transcription-related nuclear events irrespectively of agnogene presence.

To further explore the functions of differentially expressed protein-coding RNAs, the subcellular localization of their protein products was also assessed using GeneCards data. Those with nuclear expression scores of 4 or 5 were classified as nuclear proteins. Among the genes that were uniquely altered in WT/AVP231 and Dl-Mt/VP231, 49% and 42%, respectively, were predicted to encode nuclear proteins, whereas among shared genes, 37% were (Supplementary Dataset S2B). However, although JCV capsid proteins are more restrictively localized to PML-NBs in the presence of the agnogene, the dynamic reorganization of host nuclear proteins was not so pronounced.

### 3.5. GO Enrichment Analysis Using DAVID

Next, GO enrichment analysis was conducted using the DAVID platform to investigate the biological significance of the RNA transcriptomic variations. In WT/AVP231-transfected cells, 416 differentially expressed non-coding RNAs and protein-coding RNAs were identified (Supplementary Dataset S1). Among them, 281 genes with functional annotation were included in the DAVID-based GO analysis, which yielded 191 functional clusters, with the top-ranked cluster (enrichment score = 3.18) involving chromatin organization (GO:0006325), chromatin remodeling (GO:006338), and histone-modifying activity (GO:0140993).

**Figure 5:**
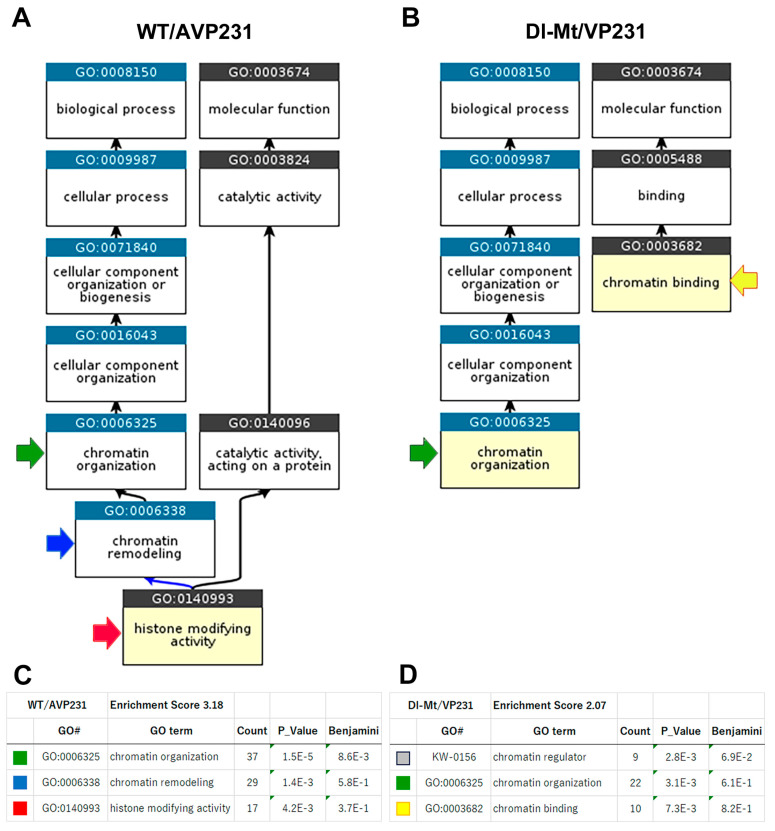
Enriched GO term hierarchy of differentially expressed genes. (**A**,**C**) Functional annotation of genes altered by WT/AVP231 transfection revealed clusters related to chromatin organization (GO:0006325, green), chromatin remodeling (GO:0006338, blue), and histone-modifying activity (GO:0140993, red). (**B**,**D**) In Dl-Mt/VP231-transfected cells, the highest-ranked clusters also involved chromatin-related functions, albeit with lower enrichment scores. The comparison suggests the importance of the agnogene for chromatin dynamics.

In Dl-Mt/VP231-transfected cells, 274 differentially expressed transcripts were identified, and 187 genes were eligible for DAVID-based GO analysis. The top cluster (enrichment score = 2.07) included chromatin binding (GO:0003682), chromatin organization (GO:0006325), and the UniProt keyword “chromatin regulator”. The results highlighted nuclear functions and a potential agnogene function regarding chromatin regulatory mechanisms. GO hierarchies and enrichment scores are presented in Figure 5.

### 3.6. Agnogene Modulates the Dynamics of Epigenetic Regulators

**Figure 6:**
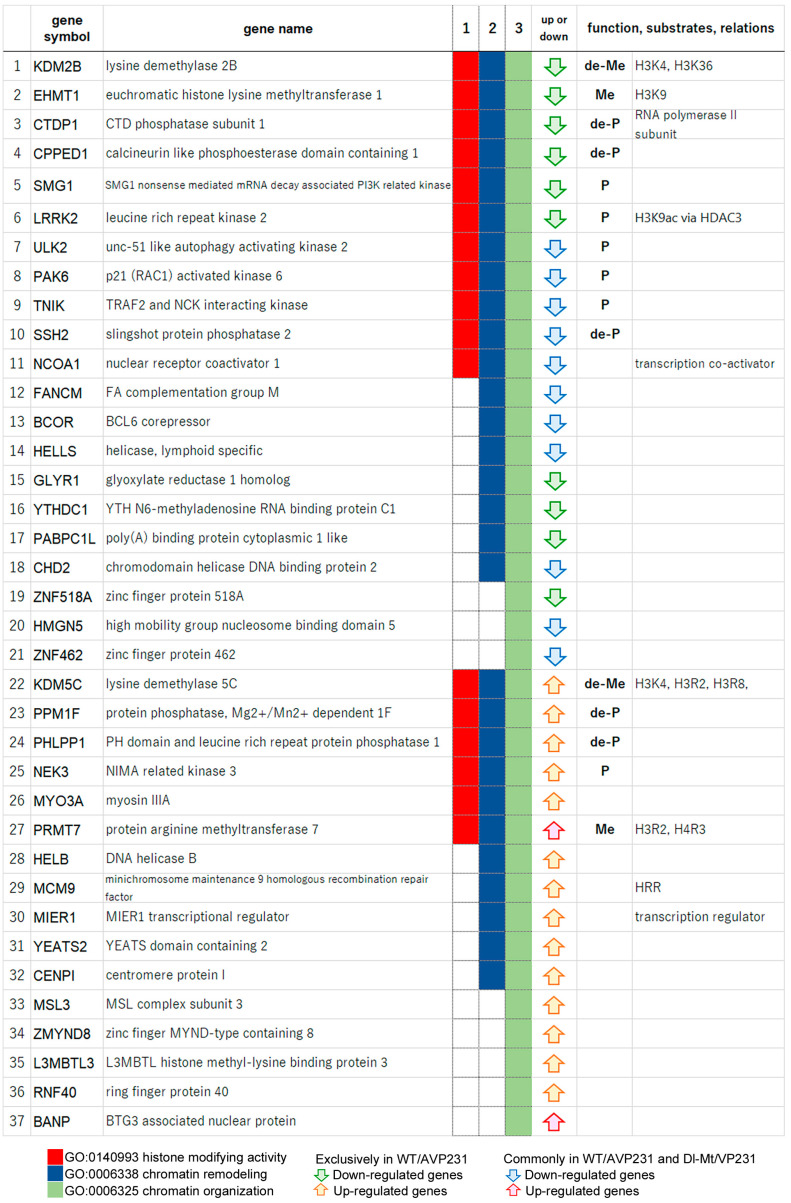
Chromatin organization genes in WT/AVP231-transfected cells. Differentially expressed genes in WT/AVP231-transfected cells included the 37 chromatin organization genes (GO:0006325, green), which contained 17 genes with histone-modifying activity (GO:0140993, red). These included kinases (e.g., SMG1, LRRK2), phosphatases (e.g., CTDP1), methyltransferases (EHMT1, PRMT7), and demethylases (KDM2B, KDM5C). The agnogene appeared to regulate a wide spectrum of chromatin-associated enzymes.

To further elucidate how the agnogene influences transcription processes in the nucleus, particularly via epigenetic regulation, we listed the genes clustered within the highest enrichment scores. In cells transfected with WT/AVP231, 37 genes associated with chromatin organization (GO:0006325) were identified, including 17 involved in histone modification activity (GO:0140993) (Figure 6). These included six kinases (SMG1, LRRK2, ULK2, PAK6, TNIK, MEK3), five phosphatases (CTDP1, CPPED1, SSH2, PPM1F, PHLPP1), two methyltransferases (EHMT1, PRMT7), and two demethylases (KDM2B, KDM5C). These enzymes play key roles in chromatin state regulation, indicating that the agnogene functions to regulate transcriptional landscapes via histone modification and chromatin-associated proteins. Notably, 11 of these 17 genes were differentially expressed exclusively in WT/AVP231-transfected cells, suggesting the critical role of the agnogene.

In Dl-Mt/VP231-transfected cells, 21 genes associated with chromatin organization (GO:0006325) were differentially expressed, including 16 downregulated and five upregulated genes (Supplementary Dataset S3). Among the downregulated ones were three kinases (PAK6, ULK2, RPS6KB1), one phosphatase (SSH2), and one demethylase (KDM6B). While KDM6B and RPS6KB1 were not detected in WT/AVP231-transfected cells, the remaining three genes (SSH2, PAK6, ULK2) were altered both in the presence and in the absence of the agnogene. Thus, some chromatin-related genes (e.g., SSH2, PAK6, ULK2) changed in the absence of the agnogene, indicating the partial involvement of JCV capsid proteins.

**Figure 7:**
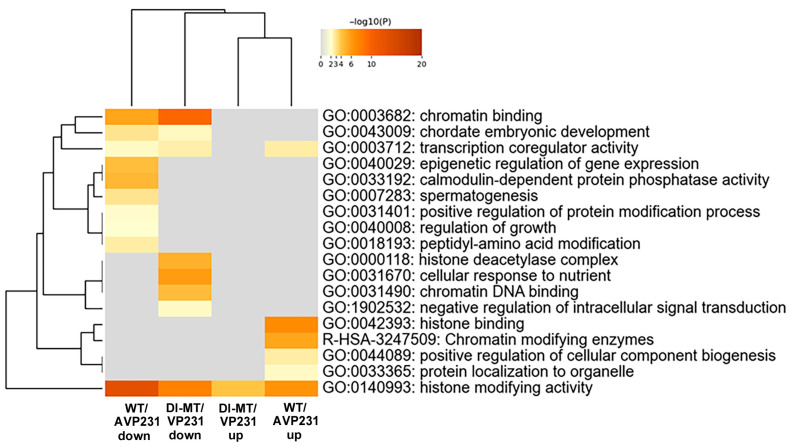
Comparative analysis of chromatin-related genes. Comparative functional annotation of genes altered in WT/AVP231- and Dl-Mt/VP231-transfected cells using Metascape. Genes involved in chromatin remodeling and histone modification were predominantly upregulated in WT/AVP231 cells and downregulated in Dl-Mt/VP231 cells, suggesting a potential role of the agnogene in nuclear regulation.

For further investigation of the exclusive roles of the agnogene and for chromatin- and histone-associated transcriptional regulation, we compared the functional annotation of the 37 altered genes in WT/AVP231-transfected cells (listed in Figure 6) and the 21 altered genes in Dl-Mt/VP231-transfected cells (listed in Supplementary Dataset S3) using a multiple-gene-list comparison function of Metascape (Figure 7). The genes with histone-modifying activity (GO:0140993) were both up- and downregulated in the presence and absence of the agnogene. However, genes associated with nuclear functions—such as chromatin remodeling and histone modification (GO:0000118, GO:0031490, GO:0042393, GO:0140993)—were predominantly upregulated in WT/AVP231-transfected cells and downregulated in Dl-Mt/VP231-transfected cells. These consistent patterns support the agnogene’s role in modulating chromatin and histone, which may eventually induce cell death. However, the overall effect of the agnogene on transcriptional activity remains inconclusive.

### 3.7. Diploidy of JCV-Infected Human Glial Cells

Finally, the number of chromosomes was examined in JCV-infected cells of human brain tissue. We previously described that JCV-infected cells enlarge their nuclei during the transition from the S to the G2 phase of the cell cycle [11]. In this process, PML-NBs also grow and reach their peak size in the G2 phase. Glia-like cells with nuclear enlargement were positive for MIB-1 (Ki-67), indicating cell cycle activation, and were found to express cyclin A, a marker of the G2 phase. However, JCV-infected human glia-like cells with enlarged nuclei lacked expression of the M-phage marker PHH3, and no evidence of mitotic division was observed as previously presented (for details, see Figure 2 and Figure 3 in the previous report [11]).

**Figure 8:**
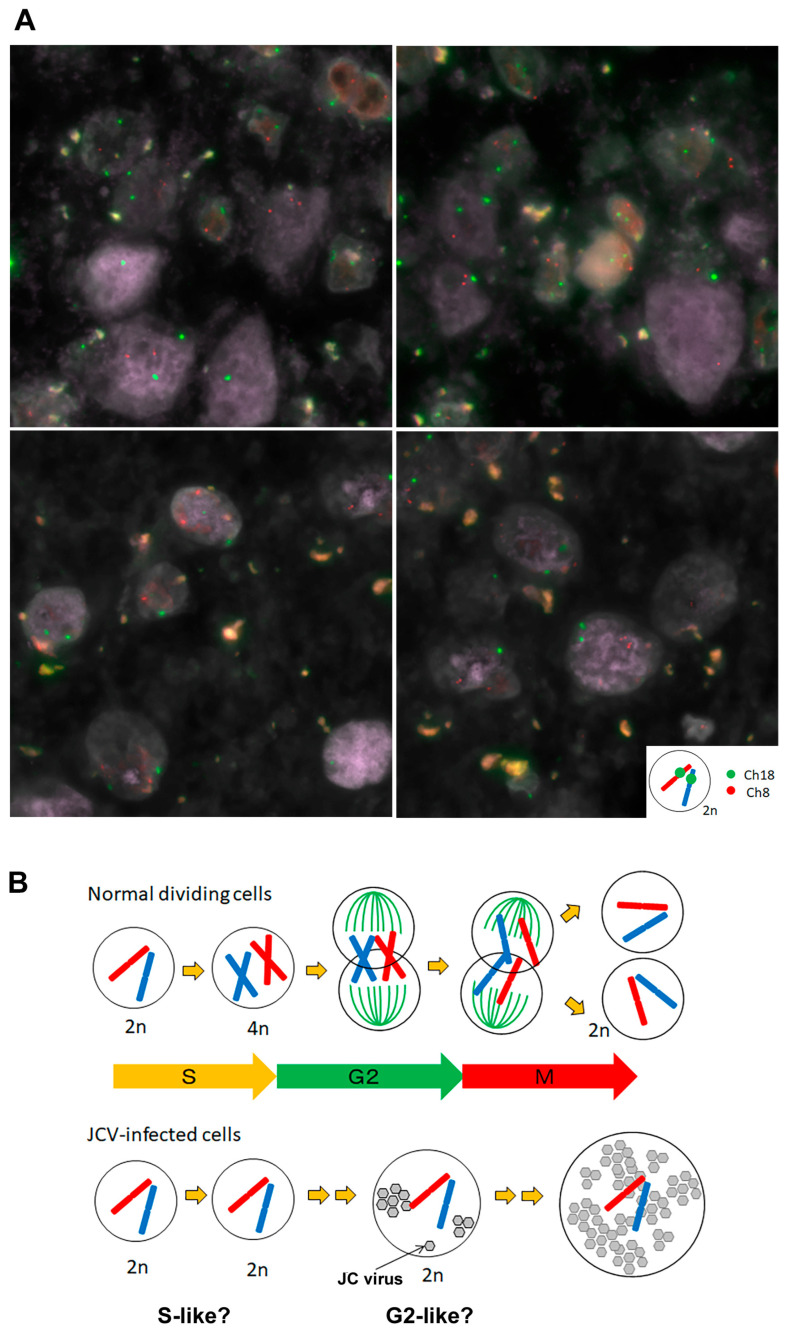
Chromosomal ploidy in JCV-infected glial cells. (**A**) Three-color FISH analysis of JCV-infected glial cells from a human brain revealed diploid chromosomal status. JCV DNA was labeled with Cy2, chromosome 8 of the host cells with Cy3, and chromosome 18 with Cy5. All the cells displayed diploidy, indicating that in JCV-infected cells, host genome duplication may not occur. (**B**) A schematic illustration of normal dividing cells and JCV-infected cells. Why M-phase transition is suppressed in JCV-infected cells remains unknown.

To explore the reason why JCV-infected cells failed to enter mitosis, we examined DNA replication status in host cells. In post-mortem human brain tissue, JCV DNA was detected using Cy2 fluorescence labeling, while human chromosomes 8 and 18 were labeled with Cy3 and Cy5, respectively, to assess chromosomal content in infected cells. All JCV-infected cells retained a diploid (2n) chromosomal configuration, and no tetraploid (4n) nuclei were identified. Although microarray analysis revealed changes in cell-division-associated genes, mitotic activity was not observed in JCV-infected cells in the human brain tissues. These findings suggest that JCV-infected cells likely enter a state of G2 arrest without proceeding to mitosis, potentially mediated by epigenetic or chromatin-based mechanisms. This highlights a limitation of the in vitro experimental model and emphasizes the need for careful interpretation when extrapolating these findings to physiological conditions (Figure 8A,B).

## 4. Discussion

### 4.1. PML-NBs and Chromatin Dynamics of Host Cells

Promyelocytic leukemia nuclear bodies (PML-NBs) were initially identified in the context of promyelocytic leukemia, which is characterized by the chromosomal translocation t (15;17) [16,17]. PML-NBs are ubiquitously present in eukaryotic nuclei and are associated with a wide range of nuclear processes, including chromatin dynamics and DNA repair [18,19]. A variety of nuclear proteins, such as PML, Sp100, p53, CBP/p300, Daxx, BLM, Pin1, HDAC7, and pRB, have been shown to localize to PML-NBs either permanently or transiently, depending on the cellular conditions.

There are increasing investigations focusing on the PML-NB functions in chromatin remodeling, particularly concerning histone H3 [20]. During the G2 phase, PML-NBs physically interact with chromatin, suggesting their involvement in transcriptional regulation [21,22]. For example, PML-NBs provide specific nuclear spaces that the de novo DNA methyltransferase DNMT3A cannot access, resulting in the steady maintenance of hypo-methylated states or open chromatin at specific genomic loci [23]. Several chromatin-related factors, including histone-modifying enzymes and histone chaperones, as well as ATRX, CBP, HDAC7, HP1, and SETDB1, have been reported to localize in PML-NBs. In the present study, among 17 differentially expressed genes with histone-modifying activity (GO:0140993), both demethylases and methyltransferases targeting lysine or arginine residues of histone H3 were included (Figure 6 and Supplementary Dataset S3). However, further investigation is required to ascertain whether these enzymes modulate the expression of neuro-specific nuclear factors, which may delineate the viral pathogenicity within the CNS.

Most recently, it was discovered that JCV exhibits epigenetic mimics, and agnoprotein interferes with nuclear heterochromatin to create virus-occupied space [24]. The authors reported that JCV agnoprotein contains, for example, motifs resembling the chromoshadow domain (CSD) and chromodomain (CD), which facilitate binding with HP1α. Agnoprotein is a phosphoprotein, and several phospho-acceptor sites have been mapped to Ser-7, Ser-11, and Thr-21 [25]. Since the genes of kinases and phosphatases were differentially expressed in the present study, it is possible that these enzymes may activate or inactivate the agnoprotein itself. Although epigenetically regulating acetylation of NF-κB p65 has been reported in previous studies of JCV [26,27], acetyltransferases were not notable in our experiments.

### 4.2. PML-NBs and JCV Agnogene/Agnoprotein

A variety of DNA viruses, including adenoviruses, papillomaviruses, polyomaviruses, Epstein–Barr Virus, and hepatitis B virus, interact with PML-NBs. However, the function of these bodies in infection differs depending on the viruses [28,29]: for some, PML-NBs possess intrinsic antiviral properties, while for others, they support viral replication. This also seems true in JCV infection: some researchers emphasized the significance of PML-NBs in anti-viral response [30], while we demonstrated that they play a supportive role in JCV capsid assembly [8,9]. A recent study also identified a novel JCV late protein, ORF4, which targets and reorganizes PML-NBs [31]. Our present study extends these observations by demonstrating that the JCV agnogene modulates chromatin- and histone-associated pathways via PML-NBs. Altered expression of enzymes such as EHMT1, PRMT7, KDM5C, and KDM6B supports a mechanistic link between viral particle assembly and host nuclear regulation.

The JCV agnoprotein is multifunctional; it is involved in DNA replication, repair and apoptosis, and it also deregulates cell cycle progression, leading to accumulation in the G2/M phase [25,32]. Interestingly, since PML-NBs serve as sites for nuclear events such as DNA repair, apoptosis, and cell cycle regulation, the diverse functions of agnoprotein appear to correspond closely with those of PML-NBs. Indeed, JCV agnoprotein has been reported to interact with cellar proteins, including p53 [33], PCNA [34], and HP1a [35], which are known to be localized to PML-NBs, indicating a strong association between agnopr.

### 4.3. Diploidy of JCV-Infected Cells in Human Brain Tissue

Finally, we have shown that JCV-infected cells with enlarged nuclei retain a diploid chromosomal complement. In a previous study, JCV-infected cells were analyzed morphometrically, and nuclear size was found to correlate with the cell-cycle state. Enlarged nuclei exhibited cyclin A expression, indicating a G2-like phase, and most of them contained giant PML nuclear bodies (PML-NBs), which are typically observed under pathological G2 conditions [11]. The retention of a diploid chromosomal complement in these JCV-infected cells suggests that host DNA replication did not occur and that only viral DNA may have been replicated. Although most epigenetic studies on agnoprotein have been conducted using a culture system of immortalized cells, our findings underscore the importance of verifying these mechanisms within the context of human brain tissue, where viral replication occurs without host genome duplication.

## 5. Conclusions

In conclusion, our findings suggest that the JCV agnogene may influence host nuclear transcriptional regulation by altering the expression of chromatin-related genes, potentially in connection with PML-NB-associated mechanisms. Although chromatin-associated transcriptomic changes were observed, JCV-infected glial cells in human brain tissue retained a diploid chromosomal status, indicating that viral replication occurs without host genome duplication. These results highlight the complexity of epigenetic interplay between viral and host factors and underscore the importance of validating in vitro findings within physiologically relevant human tissue contexts. Still, the neuro-specific pathogenicity of JCV remains unclear.

## Data Availability

The original contributions presented in this study are included in the article/Supplementary Material. Further inquiries can be directed to the corresponding author(s).

## Supplementary Materials

The following supporting information can be downloaded at https://www.mdpi.com/article/10.3390/v17101399/s1, Dataset S1: The number of differentially expressed non-coding RNAs and protein-coding RNAs in cells transfected with WT/AVP231 or Dl-Mt/VP231. Dataset S2: Three hundred and forty differentially expressed protein-coding RNAs: nuclear protein vs. non-nuclear protein. Dataset S3: Twenty-one chromatin organization genes differentially expressed in Dl-Mt/VP231-transfected cells.

## Abbreviations

JCV	JC virus
PML	Promyelocytic leukemia
PML-NBs	Promyelocytic leukemia nuclear bodies
CNS	Central nervous system
VLP	Virus-like particle
GO	Gene Ontology
