# Supplementary figures and images for: JC Virus Agnogene Regulates Histone-Modifying Enzymes via PML-NBs: Transcriptomics in VLP-Expressing Cells
Source: Viruses. 2025 Oct 21;17(10):1399. doi: 10.3390/v17101399 (PMC12567619; doi:10.3390/v17101399)

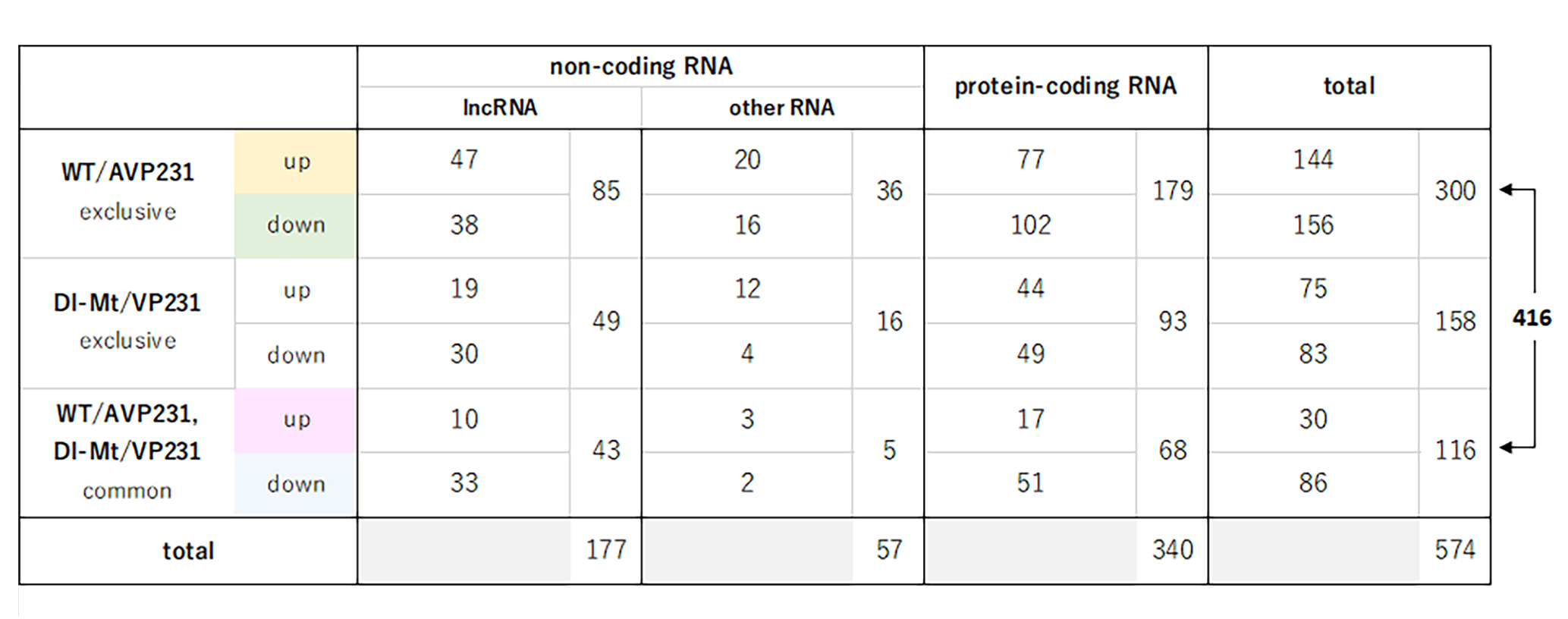

Supplement: Supplementary file 1 [file viruses-17-01399-s001.zip › Fig. Supplementary data S1.tif]

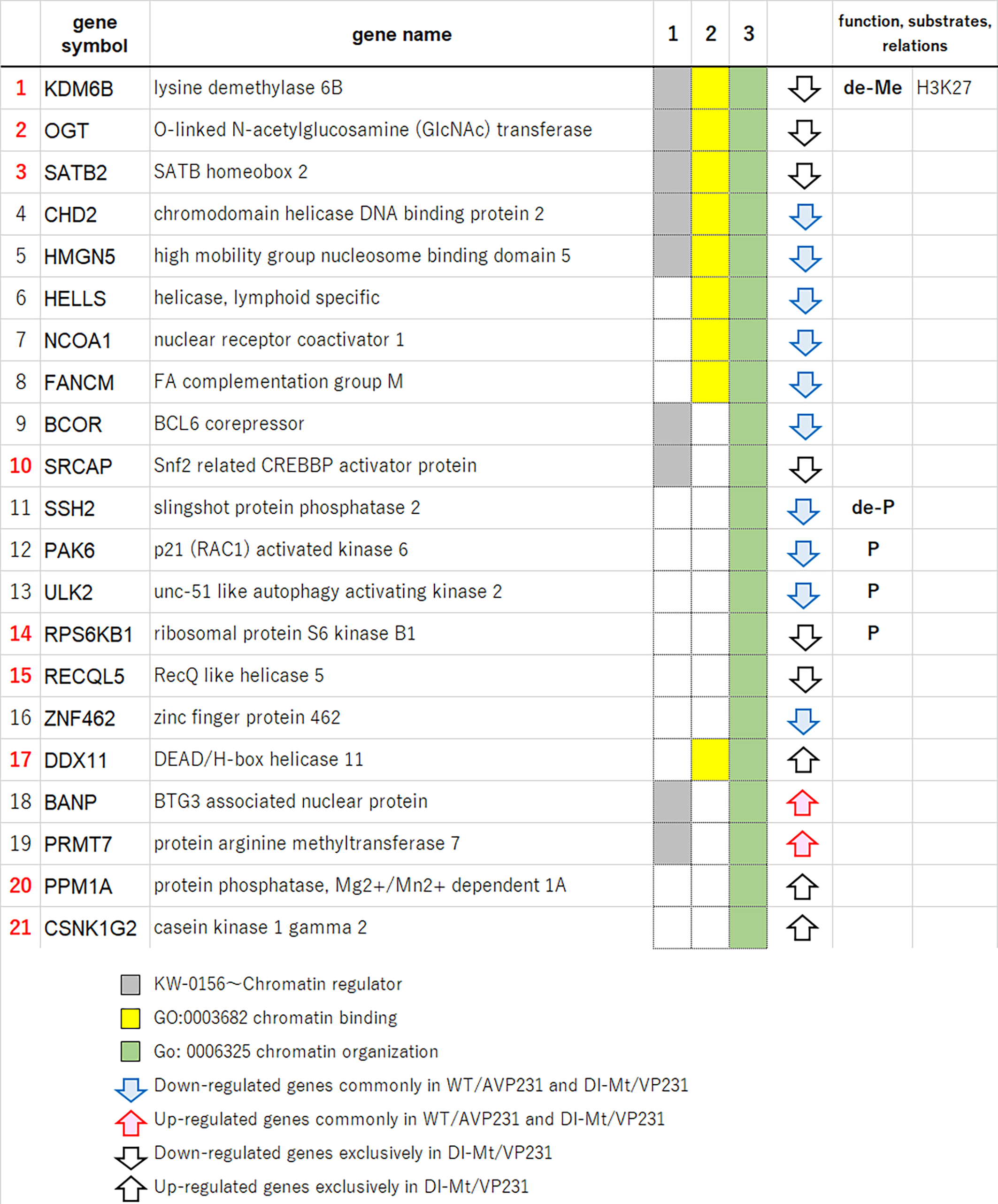

Supplement: Supplementary file 1 [file viruses-17-01399-s001.zip › Fig. Supplementary data S3 (Rev).tif]

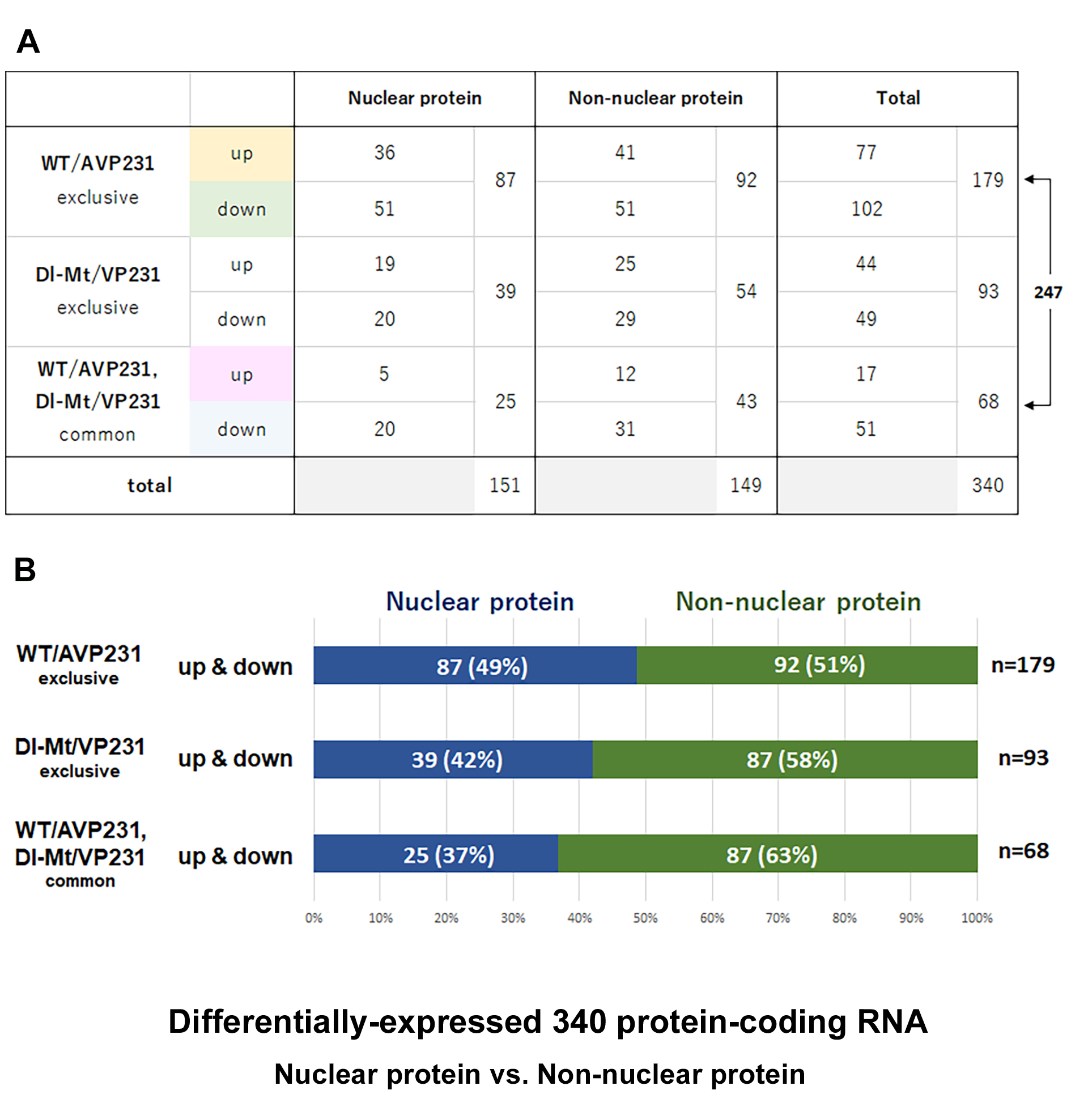

Supplement: Supplementary file 1 [file viruses-17-01399-s001.zip › viruses-3841497-20251005-Fig. Supplementary data S2.tif]
